# Onabotulinumtoxin-A treatment in Greek patients with chronic migraine

**DOI:** 10.1186/s10194-016-0676-z

**Published:** 2016-09-17

**Authors:** Michail Vikelis, Andreas A. Argyriou, Emmanouil V. Dermitzakis, Konstantinos C. Spingos, Dimos D. Mitsikostas

**Affiliations:** 1 Headache Clinic, Mediterraneo Hospital, Glyfada, Greece; 2 Glyfada Headache Clinic, 8 Lazaraki Str, Glyfada, 16675 Greece; 3 Headache Outpatient Clinic, 1st Department of Neurology, National and Kapodistrian University of Athens, Athens, Greece; 4 Neurology Department of the Saint Andrew’s State General Hospital of Patras, Patras, Greece; 5 Department of Neurology, “Geniki Kliniki” Euromedica, Thessaloniki, Greece; 6 Corfu Headache Clinic, Corfu, Greece

**Keywords:** Migraine, Chronic migraine, Treatment, Prevention, Prophylaxis, Onabotulinumtoxin-A

## Abstract

**Background:**

Chronic migraine is a disabling condition, with limited treatment options. We conducted an open label, single arm, prospective clinical trial, to assess the efficacy and safety of onabotulinumtoxin-A in Greek patients with chronic migraine. Since recent evidence suggests that a meaningful clinical response may be delayed until after a third onabotulinumtoxin-A administration, we aimed at assessing outcomes at this time point.

**Methods:**

A total of 119 patients with CM, scheduled to be treated with Onabotulinumtoxin-A (Botox ®) every 3 months, according to the approved indication and standard clinical practice, were prospectively enrolled. Data documenting changes from baseline (T0—trimester before Onabotulinumtoxin-A first administration) to the period after its third administration (T3) in (i) mean number of monthly headache days (ii) migraine severity as expressed by the mean number of days with peak headache intensity of >4/10 in a 0–10 numerical scale, and (iii) mean number of days with use of any acute headache medication, were collected from patients’ headache diaries at each visit.

**Results:**

Of the 119 patients, a total of 81 received 3 courses of onabotulinumtoxin-A and were included in the efficacy population. In those 81 patients, there was a significant decrease in mean headache days/month between T0 and T3 (21.3 ± 5.4 vs 7.7 ± 4.8; *P* < 0.001); a significant decrease in days with peak headache intensity of >4/10 (11.9 ± 5.5 vs 3.7 ± 3.3; *P* < 0.001) and finally, the change in days using acute headache medications per month between was also significant (16.2 ± 7.8 vs 5.2 ± 4.3; *P* < 0.001). Adverse events were few and of non- serious nature.

**Conclusion:**

Our results strongly support the use of onabotulinumtoxin-A for the prophylaxis of CM, as this intervention proved effective, safe and well tolerated in our cohort of Greek patients.

## Background

Migraine is the 3^rd^ most prevalent medical disorder and ranks among the leading causes of all disease-associated disability worldwide [1, 2]. Based on its frequency, migraine is subdivided into two forms, i.e., episodic (less than 15 days monthly) and chronic migraine (more than 15 headache days monthly, of which at least 8 are of migrainous type or respond to migraine-specific medication, for more than 3 months) [3]. Compared to episodic migraine, chronic migraine (CM) is associated with increased pain intensity, pain-associated symptoms, such as nausea, photophobia, phonophobia, as well as pain-related comorbidities. Moreover, patients with CM seem to have a longer average duration of headache than those with episodic migraine [4].

CM also causes greater disability in patients with increased missing household work per month, reduced productivity in household work and higher missed family activities, compared to episodic migraineurs [5]. Hence, CM has been demonstrated to significantly downgrade the health-related quality of life (HRQOL) of patients [6]. Psychiatric comorbidities seem to substantially complicate its incidence and severity [7, 8]. CM might affect up to 1 of 5 migraine patients, resulting in an estimated prevalence of about 1.4 to 2.2 % of the general population [9].

To date, the only approved treatment for CM is prophylaxis with onabotulinumtoxin A (Botox®). Its official approval in CM was given after the evaluation of its efficacy and safety in the Phase III randomized placebo-controlled identical clinical trials PREEMPT (Phase III REsearch Evaluating Migraine Prophylaxis Therapy) I and II [10, 11]. Pooled analysis of these studies, showed that onabotulinumtoxinA was effective in significantly reducing the mean frequency of headache days from baseline (week 4) to the primary endpoint at week 24, as well as in other secondary efficacy variables, including frequency of migraine, and cumulative hours of headache. Most adverse events were mild to moderate [12].

Given that recent literature contains publications reporting the outcome after administering Onabotulinumtoxin-A against CM in other South-European populations [13–17], but to our knowledge no such report exists from Greece, the aim of the current study was to explore and share our clinical experience with Onabotulinumtoxin-A in the treatment of CM in a cohort of Greek patients.

## Methods

### Study design

This study was an open-label, single-arm, prospective multicentre, clinical study, conducted in accordance with the principles of the Helsinki Declaration. Eligibility was confirmed by a protocol-specific checklist and written informed consent was obtained from each patient. Participants were enrolled from five headache centres, located in four different nodal geographic parts of Greece, including the major urban areas of Athens, Thessaloniki, Patras and the island of Corfu. The study was approved by the principal investigator’s Institutional Review Board (Mediterraneo Hospital, protocol no. 1640).

### Patient selection

The study was conducted between January 2014 and April 2016. To be eligible for enrolment, participants had to be diagnosed with CM with or without medication overuse and be scheduled to receive Onabotulinumtoxin-A, according to the approved indication and the standard clinical practice.

As a national policy and shared guideline in all Greece geographical regions, the medication is approved for chronic migraine and fully reimbursed by the public social security system for patients who inadequately responded or were intolerant to previous treatments. Hence, all of our patients were treated with Onabotulinumtoxin-A if they were diagnosed with CM and, according to current reimbursement policy in Greece, were considered as non-responders to previous preventive medications, due either to lack of efficacy or intolerance. Administration of previous orally administered migraine preventives had to be stopped at least three months prior to study entry.

Enrolled patients were also required to be adult and be able to fully understand the study information provided by the investigators. Exclusion criteria included any contraindication to Onabotulinumtoxin-A, according to the approved summary of Product’s Characteristics (SmPc) [18].

### Intervention

The study cohort consisted of patients scheduled to receive therapy with Onabotulinumtoxin-A (Botox® 100UI/fl, Allergan-Hellas), at fixed cranial and cervical sites at a fixed dose of 155 UI every three months, according to the PREEMPT paradigm and the approved Botox® SmPc [10, 18]. Administration of additional 40UI of Botox® was allowed, in line with the PREEMPT modified “follow the pain” paradigm, at the injector’s discretion [19]. No deviation from the maximum target dose (195UI) was allowed. Patients who could not tolerate the commenced fixed dose were counted as early withdrawers due to intolerability.

### Efficacy evaluation

The primary objective of our study was to evaluate the efficacy of Onabotulinumtoxin-A as expressed by the change in mean number of monthly headache days from baseline (T0—3 months period before the first Onabotulinumtoxin-A administration) to the period after its third administration (T3). Our secondary objectives included the estimation of the crude percentage of responders to intervention. Patients were classified as responders if at T3 at least 50 % reduction in headache days was achieved and further sub-classified as good responders (at least 75 % reduction in migraine days) and excellent responders (100 % reduction in migraine days—migraine free). The percentage of reduction (50, 75, 100 %) in headache days was calculated by averaging the number of days with headache per month for the three months after each treatment and comparing this with the relative number for the three months before each treatment.

Other secondary objectives included the change in migraine severity as expressed by the change in number of days with peak headache intensity of more than 4 out of 10 in a 0–10 numerical scale (moderate/severe pain), and finally the change in days with any acute headache medications used between T0 and T3. Each patient enrolled was interviewed at baseline (T0), and after each Onabotulinumtoxin-A administration. The same neurologist performed the clinical evaluation and Onabotulinumtoxin-A injections for each individual enrolled. Patients’ headache diaries were used as a source to document changes in the efficacy variables during the treatment period.

As mentioned above, our therapeutic plan was to administer at least 3 courses of onabotulinumtoxin-A to each participant before assessing efficacy (efficacy population) and thus no drop-outs were allowed earlier, unless the patients asked for their withdrawal from the intervention for any reason, including perceived lack of efficacy, adverse events, intolerance, or any other. To quantify this, we recorded the reasons for discontinuation of Onabotulinumtoxin-A administration before T3. Patients lost to follow-up for any reason consisted the Intention to Treat population (ITT) and were counted as non-responders per se.

### Safety evaluation

At each visit, patients were encouraged to report any adverse effects occurring throughout the study period either spontaneously or in response to general, non-direct questioning. Each local investigator was responsible for documenting the type and severity of overall adverse events and then categorized them for potential relationship to onabotulinumtoxin-A therapy.

### Statistical analysis

Descriptive statistics were generated for all variables. Changes in mean values of efficacy variables between T0 and T3 were assessed using the Wilcoxon rank test for paired data. All tests were two-sided and significance was set at *P* < 0.05. Statistics were performed using the SPSS for Windows (release 17.0; SPSS Inc., Chicago, IL).

## Results

A total of 119 patients (ITT population) were initially enrolled and 81 (68.1 %) of them achieved treatment with the 3^rd^ course of Onabotulinumtoxin-A, thus being included in the efficacy population analysis. A total of 38 subjects asked their withdrawal from the protocol before T3 for various reasons. Table 1 describes in detail the reasons accounting for early treatment discontinuation in those 38 cases. Noteworthy, and as outlined in Table 1, there were 8 cases that dropped out early due to “perceived good response”. Among those 8 patients, 3 returned with a relapse within 1 to 6 months after their withdrawal, 3 were lost to follow up and 2 remained in significant remission.

**Table 1:** Reasons accounting for early drop-outs (*n* = 38), before the administration of the 3^rd^ Onabotulinumtoxin-A session

Reasons of early withdrawal	N (%)
Perceived lack of efficacy by patients at T1 or T2 administration	19 (50 %)
Patients significantly improved at T1 or T2 and perceived that no additional sessions were needed	8 (21 %)
Lost to follow-up	7 (18.3 %)
Financial limitations	2 (5.3 %)
Intolerance to intervention	2 (5.3 %)

T1, T2: Trimesters after the 1^st^ and 2^nd^ Onabotulinumtoxin-A session, respectively

The 81 patients that attained the 3^rd^ course of treatment were 8 males (9.9 %) and 73 females (90.1 %) with a mean age of 43.5 ± 9.8 (range: 21–75) years. Patients had failed of a mean number of 2.9 ± 1.3 (range: 1–7) previous medications, including, on a per case basis, flunarizine, valproic acid, topiramate, propranolol and amitriptyline. A total of 39/81 (48.1 %) patients had coexistent medication overuse headache (MOH) at T0, according to ICHD-III beta criteria [3]. Psychiatric comorbidities, diagnosed according to the Diagnostic and Statistical Manual of Mental Disorders, Fifth Edition [20], were common, being evident in 52/81 patients (64.2 %). Of those 52 patients, 19 (23.5 %) had anxiety disorders, 16 (19.8 %) had depression, 14 (17.3 %) and 3 (3.7 %) had bipolar disorder. Treatment, if needed, was at their psychiatrists’ discretion, but it was recommended the use of a stable dose during the study’s period.

The analysis of response variables in the efficacy population (*n* = 81) showed that there was a significant decrease in mean monthly headache days between T0 and T3 (21.3 ± 5.4—range: 15–30 vs 7.7 ± 4.8—range: 2–19; *P* < 0.001). A total of 65/81 (80.2 %) patients were classified as responders, because they achieved response either at 50 % (*n* = 20; 24.7 %) or at 75 % (*n* = 45; 55.6 %). The remaining 16 patients (19.8 %) were considered as non-responders due to lack of efficacy at T3 (response less than 50 %), although 6 of them (37.5 % of non responders) achieved a 30 % reduction in headache days.

On an ITT basis, the analysis of results also favoured the intervention with Onabotulinumtoxin-A, as the majority of patients in total group (65/119; 54.6 %) experienced remission of at least 50 %. The percentage of first time responders after the third Onabotulinumtoxin-A course was 9.2 %, as 6/65 patients had not responded to first two sessions and responded to the third.

In addition, a significant decrease in migraine severity, as expressed by the change in the number of monthly days with peak headache intensity of more that 4 (moderate/severe pain) in a 0–10 numerical scale was noted between T0 and T3 (11.9 ± 5.5—range: 4–30 vs 3.7 ± 3.3—range: 0–18; *P* < 0.001). Finally, the change in days using acute headache medications per month between T0 and T3 was also significant (16.2 ± 7.8—range: 5–30 vs 5.2 ± 4.3—range: 0–19; *P* < 0.001). Changes in all efficacy variables between T0 and T3 (mean and median values) are summarised in Table 2.

**Table 2:** Changes in efficacy variables from baseline (T0—trimester before initiation of therapy) to the trimester after a 3^rd^ administration of Onabotulinumtoxin-A (Botox ®) (T3) in 81 patients comprising the efficacy population

	T0	T3	
Efficacy variables	Mean ± SD Range Median	Mean ± SD) Range Median	*P* value
Headache days/month	21.3 ± 5.4	7.7 ± 4.8	
	15–30	2–19	*P* < 0.001
	20	6	
Number of days with peak	11.9 ± 5.5	3.7 ± 3.3	
headache intensity of more than 4/10, /month	4–30	0–18	*P* < 0.001
	10	3	
Days with any acute headache medication/month	16.2 ± 7.8 5–30 14	5.2 ± 4.3 0–19 4	*P* < 0.001

Subgroup analysis of patients with coexistent MOH (39/81) showed that Onabotulinumtoxin-A treatment significantly reduced the mean monthly headache days at T3 compared with T0 (24.4 ± 5.4—range: 16–30 vs 10.7 ± 4.5—range: 2–19; *P* < 0.001). Amongst patients with MOH, 29/39 (74.4 %) patients were classified as responders, because they achieved response either at 50 % (*n* = 20; 51.3 %) or at 75 % (*n* = 9; 23.1 %). In those MOH patients, there was a significant reduction in days using acute headache medications per month between T0 and T3 (23.0 ± 5.5 vs 7.9 ± 4.4; *P* < 0.001).

There were few reported events of side effects, which were transient and not severe enough to justify treatment discontinuation. We recorded side effects at the following rates: wheals in the injection site (*n* = 5; 6.2 %), mild ptosis (*n* = 5; 6.2 %), lateral eyebrow elevation (*n* = 3; 3.7 %), and shoulder and/or neck pain (*n* = 3; 3.7 %). Overall, onabotulinumtoxin-A treatment was documented to be generally safe and based on just 2 cases of early withdrawal from intervention before T3 due to intolerability (neck pain in both cases), it was also proven to be well tolerated.

## Discussion

The mode of action of Onabotulinumtoxin-A in CM is not yet completely understood. It is perceived to act by interrupting peripheral nociceptor sensitisation and, subsequently, central sensitisation, which are among the central neurophysiological events of individual migraine attacks and, when often repeated, are considered to be key factors in migraine chronification [21, 22].

Since its formal approval by the official authorities, Onabotulinumtoxin-A has reached local market availability in several countries. In Greece, the use of Onabotulinumtoxin-A for CM was approved in late 2013, and as such there is a rather limited use in Greece up to date and subsequent scarcity of data on its clinical practice. To our knowledge, literature does not contain any report on the outcome of Onabotulinumtoxin-A intervention in a population of Greek patients with CM.

In the current setting, we documented a significant improvement in all efficacy variables after 3 sessions or 9 months of Onabotulinumtoxin-A exposure. Patients comprising the efficacy population (*n* = 81) obtained an 80.2 % response rate at ≥ 50 %, whereas a corresponding rate of 54.6 % was noted in the ITT population. Onabotulinumtoxin-A proved safe and well tolerated. A similar beneficial effect was observed in patients with coexistent MOH. As such, our results, overall, are in agreement with previously published studies applying similar study design in patients with or without MOH [13–17, 23–26].

To fully explore the efficacy of intervention and be able to capture patients who have not responded to initial treatment cycles, we sustained exposure to Onabotulinumtoxin-A for several months (9 months) and assessed response after the administration of its 3^rd^ course. Our decision to assess efficacy at T3 was supported by recent evidence suggesting that a meaningful clinical response to Onabotulinumtoxin-A treatment in CM may take time to occur [27, 28]. In a secondary analysis of PREEMPT, the percentage of first time responders after the third Onabotulinumtoxin-A course was 10.3 % [28]. A comparable rate (9.2 %) was observed in our study, thereby bolstering the argument that a delayed response to Onabotulinumtoxin-A therapy might not be that uncommon.

The main limitation of our study was the relatively high percentage of early drop-outs before T3 (*n* = 38; 32 %) from the initial cohort of 119 patients enrolled. Even so, the sample size of our efficacy population (*n* = 81) was larger compared to other studies with similar design [13–16]. Moreover, ITT analysis also favoured Onabotulinumtoxin-A treatment in the current setting. Other limitations include the open-label, single-arm study design we applied and the non-inclusion in the protocol of specific tools assessing disability, depression and HRQOL changes over the treatment period. In any case, we conducted and herein reported the outcome of the first prospective study that was performed to reflect real-life clinical experience with Onabotulinumtoxin-A in Greek CM patients. The long-term follow-up (12–24 months) of patients included in this study is ongoing.

## Conclusions

Treatment with Onabotulinumtoxin-A proved effective, safe and well tolerated in our setting. Further studies are warranted to explore the predictors of response to Onabotulinumtoxin-A treatment in CM patients with or without MOH. In our opinion, exploring predictors of response in essential, since new treatments for CM, such as monoclonal Abs would be available in the coming years and physicians will have to decide which treatment fits better each patient.

## Abbreviations

CM: Chronic migraine
HRQOL: Health-related quality of life
MHO: Medication overuse headache
